# Where There Are (Few) Skilled Birth Attendants

**DOI:** 10.3329/jhpn.v29i2.7812

**Published:** 2011-04

**Authors:** Ndola Prata, Paige Passano, Tami Rowen, Suzanne Bell, Julia Walsh, Malcolm Potts

**Affiliations:** ^1^Bixby Center for Population, Health and Sustainability, University of California–Berkeley, 229 University Hall, Berkeley, CA 94720-6390, USA; ^2^Bixby Center for Population, Health and Sustainability, University of California–Berkeley, G-17B University Hall, Berkeley, CA 94720-6390, USA; ^3^Department of Obstetrics, Gynecology and Reproductive Sciences, School of Medicine, University of California–San Francisco, 513 Parnassus Avenue, Box 0556, San Francisco, CA 94143, USA; ^4^Maternal and Child Health Department, School of Public Health, University of California–Berkeley, Berkeley, CA 94720-7360, USA; ^5^Maternal and Child Health and International Health, University Hall 207L MC7360, School of Public Health, University of California–Berkeley, Berkeley, CA 94720-7360, USA; ^6^Bixby Center for Population, Health and Sustainability, 207-G University Hall, University of California–Berkeley, Berkeley, CA 94720-7360, USA

**Keywords:** Cross-sectional studies, Delivery, Maternal health services, Maternal mortality, Misoprostol, Postpartum haemorrhage, Skilled birth attendants, Traditional birth attendants

## Abstract

Recent efforts to reduce maternal mortality in developing countries have focused primarily on two long-term aims: training and deploying skilled birth attendants and upgrading emergency obstetric care facilities. Given the future population-level benefits, strengthening of health systems makes excellent strategic sense but it does not address the immediate safe-delivery needs of the estimated 45 million women who are likely to deliver at home, without a skilled birth attendant. There are currently 28 countries from four major regions in which fewer than half of all births are attended by skilled birth attendants. Sixty-nine percent of maternal deaths in these four regions can be attributed to these 28 countries, despite the fact that these countries only constitute 34% of the total population in these regions. Trends documenting the change in the proportion of births accompanied by a skilled attendant in these 28 countries over the last 15-20 years offer no indication that adequate change is imminent. To rapidly reduce maternal mortality in regions where births in the home without skilled birth attendants are common, governments and community-based organizations could implement a cost-effective, complementary strategy involving health workers who are likely to be present when births in the home take place. Training community-based birth attendants in primary and secondary prevention technologies (e.g. misoprostol, family planning, measurement of blood loss, and postpartum care) will increase the chance that women in the lowest economic quintiles will also benefit from global safe motherhood efforts.

## INTRODUCTION

Recent efforts to reduce maternal mortality in developing countries have prioritized two key strate-gies: training and deploying skilled birth attendants (SBAs) and improving access to emergency obstetric care (EmOC) facilities. Both the strategies have repeatedly been shown to improve maternal and child-health outcomes for those who use these. However, these strategies are not sufficient. These do not address the safe-delivery needs of women living in remote communities, who are unlikely to be able to access either an SBA or EmOC. This flaw is critical because populations that should be at the centre of the discussion are out of reach of current interventions to reduce the maternal mortality ratio (MMR). By omitting traditional birth attendants (TBAs) and other lay birth attendants from the safe motherhood agenda, the families which rely solely on these attendants will continue to experience elevated maternal mortality and morbidity. The paper argues for the support of four community-based prevention interventions to reduce maternal mortality and morbidity in remote areas: (a) distribution of misoprostol for prevention of postpartum haemorrhage (PPH); (b) improved access to voluntary family planning; (c) simple tools to measure blood loss; and (d) better postpartum follow-up.

### Background

In 1986, the World Health Organization (WHO) estimated that over 500,000 women die from maternal causes annually, 99% occurring in developing countries. The latest inter-agency report on maternal mortality shows that the absolute numbers of maternal deaths in 2008 have declined to 358,000 but the ratio of 99% of deaths in developing countries (355,000 deaths) and only 1% of deaths in developed countries (3,000 deaths) has not changed since 1986. Regionally, sub-Saharan Africa and South Asia account for 87% of the world's maternal deaths (1). More effective strategies are required in countries where women remain at the highest risk.

## COMMUNITY-BASED PREVENTION OF MATERNAL MORTALITY

To reduce mortality in areas where most women do not receive professional help during childbirth, macro-level barriers that jeopardize maternal health (i.e. low levels of awareness, high cost of services, and inequitable distribution of SBAs) must be addressed. Creative strategies (such as output-based assistance in East Africa and Southeast Asia, cash incentives for deliveries in hospitals in India, and training and deployment of community midwives in Afghanistan) demonstrate a considerable promise as a means to increase access to maternal health services for the poor (2-6). However, large regions exist without effective maternal mortality-reduction strategies in place, often as a result of weak infrastructure and limited political commitment. Until an adequate coverage of the most effective and appropriate strategies is achieved, community-based interventions could promote maternal health and avert unnecessary mortality by training and supporting active community-based birth attendants to provide the four aforementioned low-cost interventions.

## STRENGTHENING OF HEALTH SYSTEMS: ESSENTIAL BUT INSUFFICIENT

Table 1 highlights four regions with high MMR, focusing on the 28 countries in which less than 50% of births are attended by SBAs. Each country contains large, underserved rural areas where women traditionally give birth in the home. The rural population of the 28 countries ranges from 44% to 90% (7). With high rates of poverty, great distances to facilities, and inadequate transportation, serious complications all too often lead to the death of a mother and/or the newborn. In poor countries, stark health inequities exist between urban and rural areas: the MMR estimate for urban areas is 447 per 100,000 livebirths [95% confidence interval (CI) 384-517] compared to 640 per 100,000 livebirths in rural areas (95% CI 590-630). In places such as Afghanistan, the difference is even more dramatic: the MMR in the capital city of Kabul is 418 per 100,000 livebirths compared to 6,507 per 100,000 livebirths in the remote rural district of Ragh (8).

**Table 1.** Countries where less than 50% of births are attended by skilled birth attendants

UNFPA region	Countries
Sub-Saharan Africa	Burundi, Chad, Eritrea, Ethiopia, Guinea, Guinea-Bissau, Kenya, Mali, Mozambique, Niger, Nigeria, Sierra Leone, Somalia, Tanzania, Uganda, and Zambia
Asia and the Pacific	Afghanistan, Bangladesh, Cambodia, India, Laos, Nepal, Pakistan, Papua New Guinea, and Timor-Leste
Arab States (Middle East and North Africa)	Yemen
Latin America and Caribbean	Guatemala and Haiti

Source: Measure DHS 2010 (9).

UNFPA=United Nations Population Fund

The average MMR in the countries listed in Table 1 is approximately 600 per 100,000 livebirths, ranging from 110 in Guatemala to 1,400 in Afghanistan. The average lifetime risk of a woman dying of maternal causes in these countries is approximately 1 in 55 (1).

Improvements in health facilities and training of SBAs tend to benefit women living in more developed areas but are not always brought to the scale necessary to reach women living in the most underdeveloped areas. These women tend to have little or no education and low exposure to mass media, where many public-health campaigns take place (9). As access to education and linkages with the existing infrastructure improve, increased awareness about safer childbirth strengthens the demand for skilled care and better-quality health facilities.

Some countries and regions, especially those where access to family planning has slowed the rapid population growth, have made measurable progress towards improving access to SBAs. Sri Lanka, Thailand, Iran, and Malaysia have prioritized the training and mobilization of SBAs and have found ways to make them accessible in rural areas (10). Afghanistan increased the number of SBAs from 500 to over 1,000 between 2002 and 2007 (2, 11) but the 21 midwifery schools will need to train many more midwives to meet the demands of over a million births a year. The United States Agency for International Development estimates that Afghanistan will need up to 5,000 midwives to cover the needs of its current population (12).

Global trends do show an overall increase in the use of SBAs: in all countries not listed in Table 1, over 50% of childbirths already occur with the assistance of an SBA. This paper aims to draw attention to the countries that are listed in Table 1, where urgent attention is required because the use of SBAs is low and the MMR remains high, largely as a result of political, socioeconomic, cultural and topographical barriers to timely access to EmOC (9). Access to SBAs and EmOC are perhaps the most critical factors in the decline of MMR but are not the only contributing factor. Sri Lanka, Nicaragua, and Thailand—three countries which made excellent strides in SBA deployment in recent years—also attained contraceptive prevalence rates of 68%, 72%, and 77% respectively (13). In the case of Bangladesh, the MMR has declined steadi-ly despite the low coverage (18%) of SBA-attended births (9). A study analyzing the factors contributing to the decline of MMR in Matlab, Bangladesh, hypothesized that improvements in participation of girls in school and a strong, government-backed family-planning programme that enabled women to avoid unsafe abortions (through menstrual regulation) played key roles in both fertility decline and MMR decline observed in that region (14). Among the countries in Table 1, access to family planning remains a major challenge. This is clearly reflected in population growth rates: with the exception of a few countries in South and Southeast Asia, most countries in Table 1 have growth rates of over 2.0%, which means the population-size would double within 35 years if rates were to remain constant. Of the countries highlighted in the African region, the average population growth rate is 2.8% (13).

To illustrate the importance of a renewed focus on the countries listed in Table 1, we analyzed the trends in the coverage of skilled birth attendance using data from the demographic and health surveys (DHS) with the most recent estimates on MMR from the latest inter-agency report on trends in maternal mortality (1). Table 2 reveals the total number of maternal deaths in all the countries in which less than 50% of births are attended by an SBA compared to the total number of maternal deaths in each of the four regions covered by the United Nations Population Fund. The proportion of maternal deaths that each subset of countries contributes to its respective region is shown in the 5th column. This calculation reveals the extent to which these 37 countries are driving up the MMR in their regions. It also highlights the potential to reduce the MMR by developing and implementing community-based interventions in more remote areas of these countries.

**Table 2.** Impact of specific countries on regional maternal mortality rates

UNFPA region	Regional MMR*	No. of maternal deathsper region*	No. of maternal deaths*, † (from countries where fewer than 50% of births are attended by SBAs)	% of maternal deaths*, † (from countries where 6fewer than 50% of births are attended by SBAs)	% of regional population‡ (from countries where <50% of births are attended by SBAs)	No. of countries with less than 50% SBA attendance†
Sub-Saharan Africa	630	189,000	130,070	69	56	16
Asia and the Pacific	200	136,000	112,570	83	68	9
Arab States (Middle East and North Africa)	250	21,000	1,800	9	9	1
Latin America and Caribbean	85	9,200	1,300	14	4	2
Total	1,165	355,200	245,740	69	34	28

Sources:

*World Health Organization 2010 (1)

†Measure DHS 2010 (9)

‡Population Reference Bureau 2010 (13).

MMR=Maternal mortality ratio;

SBA=Skilled birth attendant;

UNFPA=United Nations Population Fund

Table 2 indicates that 69% of maternal deaths in sub-Saharan Africa can be attributed to these 16 countries where the use of SBAs is less than 50%. These 16 countries constitute 56% of the population in sub-Saharan Africa. In Asia and the Pacific, nine countries account for 83% of annual maternal deaths in the entire region, although they account for only 68% of the total population. In the Arab states, the prevalence of SBAs is high with the exception of Yemen where 64% of women deliver at home without professional assistance. Yemen accounts for 9% of maternal deaths in the region which comprises the Middle East and North Africa, and it also contains 9% of the population. The latest WHO estimate for MMR in Yemen is 210, a surprisingly low figure considering the 2000 estimate which was 850 per 100,000 livebirths (1, 15). If Yemen has actually achieved such a precipitous decline, the safe motherhood community certainly needs to know how such a remarkable feat could have been achieved. In Latin America and the Cari-bbean, there are only two countries (Guatemala and Haiti) in which less than half of the women use SBAs. These two countries alone account for 14% of all maternal deaths in the region, although they constitute only 4% of the population. Overall, 69% of maternal deaths in these regions occur in countries where less than 50% of births are attended by SBAs, although these countries comprise only 34% of the total population. Thus, there are more than twice as many maternal deaths occurring in these countries.

### The long road ahead: achieving adequate coverage of skilled birth attendants

**Fig. 1.**
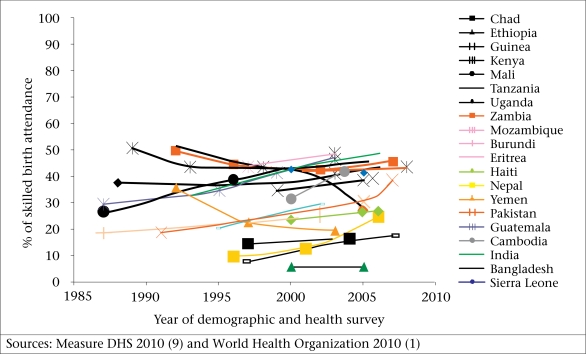
Trends in coverage of skilled birth attendants between 1987 and 2008

**Fig. 2.**
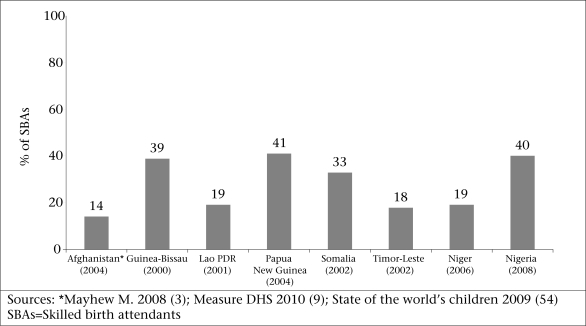
Percentage of births accompanied by skilled attendants among countries lacking reliable DHS trend data

Even with the current intensity of efforts to improve access to quality delivery care, the proportion of births attended by an SBA is likely to change very slowly if the past trends are any indication. Insufficient change has occurred over the last 15 years (Fig. 1). Figure 1 reveals the trends in the use of SBAs in 22 of the 28 countries discussed in this paper. Eight countries (Fig. 2) were excluded for lack of reliable data on trends.

Several South Asian countries have made impressive strides towards increasing the use of SBAs, including Nepal, Pakistan, India, and Bangladesh. In Africa, Eritrea, Mali, and Mozambique show a steady rise. Cambodia and Guatemala have also made impressive gains. However, most trends are more or less flat: approximately 50% of the countries tracked have shown a negligible change, no change, or even a reduction in the proportion of women accessing SBAs.

Table 3 demonstrates the enormous challenge the governments face in achieving adequate density of SBAs in the context of high total fertility rates (TFR). The countries in Table 3 are ranked from a high TFR of 7.2 to a low of 2.8. Of the 30 countries listed, 17 have a difficult road ahead, with a total population of over 10 million and an MMR of over 500 per 100,000 livebirths. In South Asia, India, Pakistan, and Bangladesh have managed to bring their MMR below 350 but will continue to face the challenge of ensuring adequate coverage of key interventions, with total populations between 164 million and 1.2 billion.

**Table 3.** Challenge of fertility and population growth in SBA deployment

Country	TFR (2007)*	Total population (million) (mid-2010)†	Maternal mortality ratio‡	Density of nursing and midwifery personnel, per 10,000 population (data 2000-2009)¶
Niger	7.2	15.9	820	1
Guinea-Bissau	7.1	1.6	1,000	6
Afghanistan	7.1	29.1	1,400	5
Burundi	6.8	8.5	970	2
Timor-Leste	6.6	1.2	370	22
Uganda	6.5	33.8	430	13
Mali	6.5	15.2	830	2
Sierra Leone	6.5	5.8	970	2
Chad	6.2	11.5	1,200	3
Somalia	6.1	9.4	1,200	1
Yemen	5.5	23.6	210	7
Guinea	5.5	10.8	680	<0.5
Nigeria	5.4	158.3	840	16
Ethiopia	5.3	85.0	470	2
Zambia	5.2	13.3	470	7
Mozambique	5.2	23.4	550	3
Tanzania	5.2	45.0	790	2
Eritrea	5.1	5.2	280	6
Kenya	5.0	40.0	530	12
Guatemala	4.2	14.4	110	N/A
Papua New Guinea	3.8	6.8	250	5
Haiti	3.6	9.8	300	N/A
Pakistan	3.5	184.8	260	4
Nepal	3.3	28.0	380	5
Lao PDR	3.2	6.4	580	10
Cambodia	3.2	15.1	290	8
Bangladesh	2.9	164.4	340	3
India	2.8	1,188.8	230	13

Sources:

*State of the world's children 2009 (54);

†Population Reference Bureau 2010 (13);

‡World Health Organization 2010 (1);

¶World health statistics 2010 (18);

N/A=Data not available;

TFR=Total fertility rate

In a 2004 study of the global health workforce, Chen *et al*. concluded that the prospects of achieving sufficient coverage of maternal health interventions are limited without sufficient density of skilled workers (16). Niger, for example, has 16 million people and almost one million births a year but it has only 288 physicians and 2,115 midwives to serve the population (13, 17). In tracking progress towards the Millennium Development Goals (MDGs), improvements stated in percentages can be misleading in countries such as Niger because they can obscure the situation that is reflected by absolute numbers. Niger, with less than one doctor and just over one midwife per 10,000 people, is not alone. Countries in sub-Saharan Africa have, on average, two physicians and 11 nurses or midwifery personnel per 10,000 people, mostly clustered in urban centres; this is compared to Europe, which has 23 physicians and 68 nurses/midwifery personnel per 10,000 people (18).

Six of the 28 countries listed in Table 1 lacked sufficient DHS data to plot the trends in the use of SBAs but the most recent available point estimates showing the percentage of births attended by SBAs are displayed in Figure 2. The MMR in the countries shown in Figure 2 ranges from 250 to 1,400, with an average of 800 (1). It is not plausible that the majority of women in the six countries depicted in Figure 2 will be able to access care from skilled birth attendants in the near future. In the interim, governments, international NGOs, and local NGOs need to bring the four interventions and associated technologies mentioned above to those women without SBAs.

### How should scarce maternal health resources be spent?

Where TBAs are the only birth attendants who live among the women they serve, they should be trained in these four areas. Where other types of community-based outreach workers have been deployed, their training should also include these four interventions. In both cases, the importance of community-based birth attendance lies in their physical proximity to women who are likely to give birth outside health facilities. Community-based interventions are complementary to long-term strategies to train more SBAs. Most importantly, the four strategies listed in this paper should be integrated into existing safe motherhood efforts.

Increasing the practice of postpartum visits by community members who are knowledgeable about danger-signs can be implemented at little to no cost, with great potential for improved health outcomes. This simple practice could prevent deaths due to sepsis and secondary PPH and a large proportion of neonatal deaths resulting from hypothermia, neonatal sepsis, dehydration, or non-exclusive breastfeeding (19). In addition, postpartum visits could reduce poor maternal and neonatal health outcomes by promoting longer birth intervals through the provision of postpartum family planning (13, 20, 21).

Increased access to family planning and fewer pregnancies at the extremes of fertile life has contributed up to half the reduction in maternal mortality ratios in the Global North. Making family planning widely available in low-resource settings (and thus reducing unwanted pregnancies) has the potential to avert 32% of maternal deaths and 10% of childhood deaths in countries with high birth rates (21, 22). One of the strengths of family planning, which is a choice, not a diagnosis needing a trained health worker, is that it can be put in place even before other aspects of primary healthcare are on the ground. Social marketing and community-based distribution of family planning need to be greatly strengthened for these benefits to be realized. The current emphasis on SBAs and EmOC has largely overlooked the opportunity to train community-based birth attendants or other health workers in family-planning advice and referrals. This omission needs correction.

Debates surrounding knowledge and abilities of TBAs and the potential of empowering women to help themselves have also diverted attention from a number of underused technologies that could significantly reduce maternal mortality. Earlier efforts to train TBAs failed because there was no skill or technology that could be taught to TBAs. All that changed with community-level use of misoprostol, a low-cost, thermostable, uterotonic tablet. Several studies have demonstrated that TBAs (and birthing women themselves) can administer misoprostol effectively and safely to reduce the incidence and severity of PPH in places where skilled attendants are not available (23-25). While non-professional birth attendants will never replace the need for well-trained, experienced SBAs able to conduct active management of the third stage of labour, non-professional birth attendants can become more effective in saving the lives of mothers and newborns if given training in best practices and offered basic materials and support in facilitating timely referral (26).

Locally-available means of gauging blood loss have also been shown to be an effective means of alerting birth attendants and families of the threshold for PPH. A field trial in Tanzania by Prata *et al.* found that trained TBAs were able to correctly identify two soaked *kangas* (women's dress fabric which can absorb approximately 500 mL of blood) as the appropriate moment to initiate transfer to a health facility (25). Similar efforts have been tested in India using a calibrated drape and are currently underway in Bangladesh, using a locally-manufactured delivery-mat invented by researchers at the International Centre for Diarrhoeal Disease Research, Bangladesh (27). Incorporating approaches such as these into a more comprehensive strategy to reduce MMR requires the active collaboration of local NGOs or organized women's groups which have the financial means to support and supply birth attendants and the women they serve with the knowledge and materials required for safer births in the home.

Debate continues concerning how limited resources can most effectively be spent to reduce the global MMR. Many have cautioned that money spent to train TBAs will divert funds from the limited pool of money needed to train and deploy SBAs (28). This concern is understandable because training and retaining SBAs is both costly and time-consuming but training TBAs is not. Midwives require 8-12 years of basic education, followed by 2-3 years of midwifery training. In Malawi, pre-service training of a single nurse-midwife costs the Government between US$ 4,000 and $ 26,000 (depending on level of certification), and government investments in medical personnel offer no guarantee of return on investment (29). After certification, medical providers tend to migrate to countries offering better salaries, working conditions, and educational opportunities for their children. This makes further government investment necessary, including financial incentives to motivate providers to remain in areas where the need is the greatest. The 2005 World Health Report of the WHO estimates that, by 2030, approximately 700,000 SBAs will be required to ensure the full coverage of SBAs worldwide: 330,000 newly-trained SBAs, along with 370,000 replacements for those who will be lost to attrition (30).

If integrated with the existing programmes, training TBAs and women themselves in safe delivery, including misoprostol, need not be costly. A 2007 cost-effectiveness study determined the cost per trainee (including teacher and materials) to train TBAs in misoprostol for the treatment of PPH ranged from US$ 3 to $ 17 per trainee in Afghanistan (1999) and India (2005). To quantify the potential savings to the health system, two theoretical cohorts of 10,000 women were modelled which concluded that the misoprostol strategy could prevent 1,647 cases of severe PPH (range 810-2,920) and save US$ 115,335 in costs of referral, intravenous therapy, and transfusions (range US$ 13,991-$ 1,563,593) per 10,000 births (31). A recent Cochrane Review called for “increased research on low cost, low technological home-based management of post-partum haemorrhage” (32). If home-based management strategies for PPH are to be implemented to scale, as can be seen in Table 3, resources cannot be limited to training SBAs; strategies will need to include traditional birth attendants (TBAs), other community members who attend births, and birthing women themselves.

As an illustrative example, take a country whose annual SBA budget is US$ 200,000. Using a conservative estimate (assuming the country used all US$ 200,000 to train people who already had a general education) that would produce approximately 20 SBAs over the next 1-3 years; this is based on the aforementioned Malawi cost-effectiveness figures (29). After the initial training, these skilled professionals would continue to require salaries and incentives to encourage them to reside in the least-connected areas. During the period while these SBAs are being trained, at-risk women will continue to deliver without the necessary support. If this country's SBA budget is increased by just 10% (US$ 20,000), this additional money could be used for training up to 6,000 TBAs within a month to enable them to use misoprostol and improve referral skills using a simple, effective means of measuring blood loss. These skills would address the most common cause of MMR worldwide (i.e. PPH) (31). In addition, TBAs could be instructed to perform postpartum visits, which could reduce mortality due to sepsis and delayed onset of PPH. A follow-up training on community-based distribution of family planning could train TBAs to deliver three basic contraceptive methods (i.e. condoms, pills, injectables) that often lie underused in health facilities (33). Countries could implement this interim strategy relatively quickly while medium and long-term solutions are still coming to fruition.

Current levels of investment, although inadequate, are expected to result in an overall increase in the number of SBAs and EmOC facilities. In the poorest countries, however, external funding will be required indefinitely. Given the current scarcity of SBAs, most will be deployed in well-equipped health facilities with large catchment areas, where professionals can assist the maximum number of women. New facilities are less likely to be built in the most remote areas, where sub-populations with the highest needs reside. A few countries, such as Sri Lanka and Malaysia, have managed to deploy SBAs to achieve high coverage in rural communities but achievability of this strategy depends on available workforce, terrain, infrastructure, and political will (34, 35). Koblinsky *et al*. point out that it is far more cost-effective to have women come to SBAs based in health facilities than to deploy SBAs to remote villages where their time would be less productive, and they would lack back-up support (36). However, it is in these remote areas that MMRs are the highest. The current prioritization of resources inadvertently places higher value on saving the lives of women who can manage to reach health facilities, and it is by no means clear that a greater proportion of women in future generations will somehow manage to reach health facilities as well.

SBAs are largely recruited and trained outside the areas that have the highest MMR. Once they are trained, they are often reluctant to work in these areas, and even if they are willing to work in these areas, deploying them there may not be the most cost-effective strategy for programmes. The complementary strategy of empowering the remote communities to help themselves costs less, can be implemented on a large scale, and does not require financial incentives to get TBAs and other birth attendants to live in their own communities. Thus, minimal investments could improve the functioning of a basic system of prenatal, delivery, and postpartum care if adequate support and supervision could be established.

### Is training TBAs worthwhile in the current context?

A meta-analysis reviewing the impact of myriad TBA training on MMR illustrated that most studies reported mixed or inconclusive findings, and many have had methodological flaws (37). In addition, the TBA interventions previously examined do not include misoprostol; without real ‘tools’, as previously mentioned, it is unrealistic to expect significant improvements. Recent evidence asserting the effectiveness of misoprostol (23-25, 38-41) offers hope for safer motherhood in rural areas, and Prata *et al*. specifically illustrated that low-skilled, community health workers are able to effectively administer misoprostol to prevent PPH (25, 42, 43).

Despite funding limitations, a few studies and meta-analyses on the topic of TBA training were published in the last few decades. A 2004 meta-analysis by Sibley and Sipe found a medium, positive association between training and TBAs’ knowledge of risk factors requiring referral, and a small, positive association between TBAs’ referral behaviour and maternal service-use but the results could not be causally attributed to the training (37). A 2005 cluster-randomized controlled trial in Pakistan, which measured the impact of training TBAs, detected a statistically significant reduction in perinatal mortality of 30% and a 26% reduction in maternal mortality, although the latter was not statistically significant (44). Sibley and Sipe conducted another comprehensive review in 2006 in which they found small, significant increases in women's use of EmOC and small, significant decreases in perinatal mortality and neonatal mortality due to improved management of birth asphyxia and pneumonia. They concluded that TBA training has contributed to improved neonatal and perinatal outcomes but a causal association between training and maternal mortality could not be established, again due to incomplete reporting (45). The review also concluded that TBA training holds promise to reduce perineonatal mortality when combined with improved health systems, a finding echoed by numerous other studies evaluating the health impacts of training at the community level (46-50). Including misoprostol in future TBA training efforts could yield positive results because of the simplicity and power of the drug itself.

### Choice of families: home or hospital?

While SBAs can attend births either in women's homes or in health facilities, their power to save maternal lives dramatically increases with appropriate back-up (i.e. medical personnel and equipment), which forms a strong argument for facility-based deliveries. Since maternal complications are often unpredictable, woman in labour and delivery will remain safest if attended by an SBA in a well-supplied and functioning health facility. A woman's next safest option would be to give birth at home attended by an experienced SBA with the necessary equipment and drugs.

Safety, however, is only one factor families consider when choosing the place of delivery. Even where modern hospitals are accessible, families do not necessarily prefer these facilities to their own homes. Despite widespread availability, less than one-third of urban women in Bangladesh in 2004 reported using an SBA. Cost of services was a deterrent to hospital delivery but cultural factors and the preferences of family members also played a significant role in the decision to give birth at home (51).

The actual capacity of health facilities to accommodate all the births of the nation must also be considered. When the MDG 5 was drafted, the question of health facility readiness was not given adequate attention. The healthcare infrastructure of many developing countries can only handle a tiny fraction of the annual deliveries. In 2002, one study estimated that Guatemalan hospitals were only prepared to manage 20% of women giving birth (52). The Government of Bangladesh is pursuing both facility-based and community-based prevention strategies, in acknowledgement of the limitations of its own obstetric units to manage the sheer number of births that occur each year.

First-hand experiences with health facilities are another reason that some families are wary of facility-based births (36). If the quality of healthcare is poor (or was poor in the recent past), community members are often well-informed of substandard conditions. Stories of rude, inattentive, or absent medical staff, inadequate stocks of drugs, and non-functioning equipment spread quickly (53). As health systems are strengthened and improved, it can only be hoped that reports of these positive changes will be circulated with the same speed and vigour.

While some concerns families have about health facilities are well-founded, exaggerated fears, reinforced by traditional beliefs, can scare families away from facility-based deliveries. Many communities fear hospitals because they are afraid of caesarean sections, or other fearful events, although their risks of such outcomes are low (52, 53). Given the natural human tendency to fear death, infertility, and invasive procedures, it is understandable that families may feel more comfortable having a woman stay home to give birth where family members will support her. Exaggerated perceptions of danger can be slowly reduced with improved communication and community outreach but the deepest-held fears will dissipate slowly.

Despite higher survival rates of mothers in childbirth, health facilities will always be hard-pressed to compete with homes in terms of comfort, familiarity, the presence of family members, and attention to valued cultural practices. As new EmOC facilities are built and the expected population to be served is estimated, factors, such as poverty, extreme weather, geographic distance, traditional belief systems, weak transportation systems, over-worked staff, and wariness of health facilities, must be realistically considered. All of the above are likely to remain formidable barriers to facility-based births in the years to come.

### Conclusions

In countries or regions where there is already a perceptible shift towards SBA and EmOC-use, it makes sense to focus investments in strengthening of health facility, which will augment the positive effects of this trend. However, the impact of these interventions should be evaluated on a country-by-country and regional basis as the impact of strengthening of health systems may be insufficient in regions with the highest MMR, where the greatest potential exists to improve maternal and newborn survival. As the struggle to increase access to quality care continues, the potential of community-based strategies must be considered seriously.

Including community-based birth attendants in the safe motherhood agenda will help get life-saving technologies and practices to women in the remote areas, who need it most. Training of these birth attendants on (a) distribution of misoprostol, (b) distribution of family-planning methods, (c) use of simple tools to measure blood loss, and (d) practice of postpartum visits has enormous potential to reduce maternal mortality and morbidity in the most underserved areas. Countries where less than 50% of births are currently attended by SBAs should strongly consider implementing these simple, low-cost strategies as an interim approach to improving maternal health while longer-term strate-gies are slowly coming to fruition: these women cannot wait.

## ACKNOWLEDGEMENTS

The authors thank Colette Auerswald for her role as a mentor to Tami Rowen and Karen Weidert and Caitlin Gerdts for their careful editing.
